# Harnessing crop health for the future

**DOI:** 10.1007/s44297-023-00004-x

**Published:** 2023-08-10

**Authors:** Kong Luen Heong, Xuexin Chen

**Affiliations:** 1 College of Agriculture and Biotechnology, Zhejiang University, Hangzhou, 310058 China; 2 State Key Laboratory of Rice Biology, and Ministry of Agricultural and Rural Affairs Laboratory of Molecular Biology of Crop Pathogens and Insects, Zhejiang University, Hangzhou, 310058 China

As the global population continues to grow, the demand for agricultural products will continue to increase, placing greater pressure on crop production systems [1, 2]. Crop health plays a vital role in sustainable agriculture by guaranteeing both food security and environmental sustainability [3]. However, crop health faces numerous challenges, including emerging diseases, invasive pests, climate change, environmental pollution and the need to reduce reliance on chemical inputs [4–6]. To address these challenges, it is essential to explore future prospects and develop innovative strategies to maintain and enhance crop health [7].

We are pleased to announce the launch of *Crop Health*, a multidisciplinary and peer-reviewed journal dedicated to publishing high-quality, cutting-edge research that contributes to the development of sustainable and resilient crop production systems. Our goal is to promote global food security and environmental sustainability. It will provide a platform to foster collaborations and exchanges of ideas among researchers, academics, and practitioners, generating advanced knowledge, better understanding, and innovative technologies for crop health management. The Editorial Board of *Crop Health* is led by two Editors-in-Chief, Professor Xuexin Chen and Professor Kong Luen Heong, with wide experiences and expertise in plant protection and agricultural biotechnology. In collaboration with a group of esteemed scientists from around the world, the journal will provide substantial breadth and depth of knowledge to the research field, ensuring a high standard of quality. The advisory board is composed of ten distinguished scientists from different continents to ensure the global scope, reach, and impact of the journal. To stay abreast of the times and innovations, the journal extends an open invitation to talented and passionate young scientists from around the world to serve as guest editors.

*Crop Health* is a Gold Open Access journal that offers free access to all its contents, enabling wider and timely dissemination of research findings. With articles freely and permanently available online immediately upon publication, they enjoy broader distribution and increased visibility compared to subscription-based content. Articles published in open access tend to receive higher citations, downloads, views, and attention. Moreover, the journal is fully sponsored by Zhejiang University and currently waives Article Processing Charges (APCs) until 2025. For more information about the Open Access policies, please visit the *Crop Health APCs* page.

The future of *Crop Health* holds immense promise for sustainable agriculture and global food security. By addressing the challenges posed by climate change, adopting integrated pest management practices, harnessing big data and digital technologies, exploring biocontrol and biotechnology, and remediating environmental pollution [8–11], we can enhance the resilience and productivity of crops. Collaboration among researchers, policymakers and farmers is essential to translate these prospects into practical solutions that can be implemented at the field level. With concerted efforts and investments in research and innovation, we can pave the way for a future where crop health management plays a vital role in sustainable agriculture, securing food production for generations to come. Collectively, we are proud to introduce *Crop Health*, the first comprehensive high-level international academic journal in the field of plant protection. We warmly welcome you to collaborate with us to make *Crop Health* a leading platform for discoveries and innovations in the field.
